# Relationship between hydrogel spacer distribution and dosimetric parameters in linear‐accelerator‐based stereotactic body radiotherapy for prostate cancer

**DOI:** 10.1002/acm2.14294

**Published:** 2024-02-06

**Authors:** Shingo Ohira, Hideomi Yamashita, Masanari Minamitani, Subaru Sawayanagi, Mami Ogita, Toshikazu Imae, Atsuto Katano, Yuki Nozawa, Takeshi Ohta, Kanabu Nawa, Teiji Nishio, Masahiko Koizumi, Keiichi Nakagawa

**Affiliations:** ^1^ Department of Comprehensive Radiation Oncology The University of Tokyo Tokyo Japan; ^2^ Department of Medical Physics and Engineering Osaka University Graduate School of Medicine Suita Japan; ^3^ Department of Radiology The University of Tokyo Hospital Tokyo Japan

**Keywords:** dose, hydrogel spacer, prostate, SBRT

## Abstract

**Purpose:**

To explore the potential of quantitative parameters of the hydrogel spacer distribution as predictors for separating the rectum from the planning target volume (PTV) in linear‐accelerator‐based stereotactic body radiotherapy (SBRT) for prostate cancer.

**Methods:**

Fifty‐five patients underwent insertion of a hydrogel spacer and were divided into groups 1 and 2 of the PTV separated from and overlapping with the rectum, respectively. Prescribed doses of 36.25–45 Gy in five fractions were delivered to the PTV. The spacer cover ratio (SCR) and hydrogel–implant quality score (HIQS) were calculated.

**Results:**

Dosimetric and quantitative parameters of the hydrogel spacer distribution were compared between the two groups. For PTV, *D*_99%_ in group 1 (*n* = 29) was significantly higher than that in group 2 (*n* = 26), and *D*_max_, *D*_0.03cc_, *D*_1cc_, and *D*_10%_ for the rectum were significantly lower in group 1 than in group 2. The SCR for prostate (89.5 ± 12.2%) in group 1 was significantly higher (*p* < 0.05) than that in group 2 (74.7 ± 10.3%). In contrast, the HIQS values did not show a significant difference between the groups. An area under the curve of 0.822 (95% confidence interval, 0.708–0.936) for the SCR was obtained with a cutoff of 93.6%, sensitivity of 62.1%, and specificity of 100%.

**Conclusions:**

The SCR seems promising to predict the separation of the rectum from the PTV in linear‐accelerator‐based SBRT for prostate cancer.

## 1 INTRODUCTION

Developments in radiotherapy, such as intensity‐modulated and volumetric modulated arc therapies, have reduced the gastrointestinal and genitourinary toxicity rates in patients with prostate cancer compared with previous techniques,^1^ and their results can be comparable to those of prostatectomy.^2^ Recently, the α/β value of prostate cancer (1.5−1.8 Gy) has been found to be lower than that of a risk organ such as the rectum (3−5 Gy),^3^, ^4^ indicating the radiobiological advantages of hypofractionated radiotherapy over conventional fractionated radiotherapy. In addition, since the COVID‐19 (coronavirus disease) pandemic, the demand for fewer irradiations has increased.^5^ These circumstances have led to the rapid implementation in clinical practice of ultrahypofractionated stereotactic body radiotherapy (SBRT), which delivers a high radiation dose in a small fraction.

SBRT provides failure‐free survival comparable to that of conventionally fractionated radiotherapy for intermediate‐to‐high‐risk prostate cancer,^6^ and the treatment approach seems to improve the efficacy and patient quality of life by reducing the frequency of medical visits. However, SBRT may cause early side effects of radiation. Insertion of a hydrogel spacer can physically separate the rectum from the prostate, drastically reducing the radiation dose to the rectum.^7^ Ogita et al. demonstrated that using a hydrogel spacer provided the dosimetric benefits of reduced rectal doses and improved patient‐reported acute bowel toxicity.^8^ Similarly, Kundu et al. reported that using a hydrogel spacer reduced the rectal radiation dose and that the incidence of acute gastrointestinal injury in the group with hydrogel spacer was significantly lower than that without spacer.^9^

Although trained physicians insert hydrogel spacers, variabilities in both the separation between the prostate and rectum and hydrogel spacer distribution are observed. Fischer‐Valuck et al. demonstrated that the asymmetrical distribution of hydrogel spacers for three axial images (midgland axial slice, 1 cm superior to midgland, and 1 cm inferior to midgland) was significantly related to the rectal dose in conventional fractionated radiotherapy.^10^ Hwang et al. demonstrated that the hydrogel volume and angle θ formed by the prostate, hydrogel, and rectum correlated with dosimetric parameters of the rectum.^11^ The assessment of hydrogel spacer distribution can contribute to appropriate insertion, but no established method has been devised to that end. Liu et al. derived the hydrogel‐implant quality score (HIQS) related to the rectal dose in low‐dose‐rate brachytherapy, and the HIQS score could account for various aspects of the hydrogel spacer distribution including hydrogel spacer volume, left‐right (LR) symmetry, superior‐inferior (SI) symmetry, and mid‐prostate spacing created by hydrogel spacer.^12^ Although the method proposed by Liu et al. is considered reasonable because it takes into account the shape of the entire hydrogel spacer distribution, there are no studies of its application to linear‐accelerator‐based SBRT for prostate cancer. In clinical practice, a more simplified method of evaluating hydrogel spacer distribution is required. The primary purpose of inserting a hydrogel spacer is separating the rectum from the irradiated volume, that is, the planning target volume (PTV) in linear‐accelerator‐based SBRT. We hypothesized that it would be important to simply distribute hydrogel spacer widely in the SI direction to prevent PTV and rectum from overlapping.

This study was aimed to compare dosimetric parameters between two groups, namely, group 1 with the PTV separated from the rectum and group 2 with the PTV overlapping with the rectum. Second, the HIQS was evaluated for distinguishing between these two groups. Finally, it was intended to explore the potential of simplified quantitative parameters of hydrogel spacer distribution as predictors for separating the rectum from the PTV in linear‐accelerator‐based SBRT for prostate cancer.

## 2 MATERIALS AND METHODS

### 2.1 Patients and simulation

This retrospective study included 55 patients with prostate cancer who underwent SBRT with or without androgen deprivation therapy. The study design and protocols were approved by the corresponding institutional review board. A hydrogel spacer (SpaceOAR system; Boston Scientific, Marlborough, MA) was inserted into the perirectal space between the prostate and rectum under transrectal ultrasound guidance.^8^ Magnetic resonance imaging (MRI) was performed approximately 1 week after hydrogel spacer placement. For computed tomography (CT) simulation, patients were scanned in the supine position, and CT images were reconstructed with a slice thickness of 1 mm. In preparation for MRI and CT acquisitions, the patients were instructed to maintain a full bladder and had the rectum emptied by receiving an enema.

### 2.2 Treatment planning

Based on MRI/CT fusion, the target organs at risk (OARs) and hydrogel spacer were delineated by radiation oncologists. The clinical target volume (CTV) for intermediate‐/high‐risk patients comprised the prostate and proximal 10/2 cm of the seminal vesicles (SVs) according to the risk classification of the National Comprehensive Cancer Network guidelines version 2.2021.^8^ The PTV was generated by adding a margin of 3 mm in the posterior direction and 5 mm in any other direction. The Monaco treatment planning system (Elekta AB, Stockholm, Sweden) was used to deliver 36.25−45 Gy in five fractions to 95% volume of the PTV every alternate weekday. For treatment planning, a multileaf collimator of 5 mm, photon beam energy of 6 MV (flattening filter free), dose calculation using the Monte Carlo method with 1% statistical uncertainty, and dose calculation grid of 2 mm were used. Dose constraints were set for the rectum, bladder, femoral head, small bowel, sigmoid colon, and penile bulb to reduce the radiation doses as much as possible. In cases where the small bowel and PTV overlapped, the radiation oncologist cut the PTV.

### 2.3 Assessment of hydrogel spacer distribution

**FIGURE 1:**
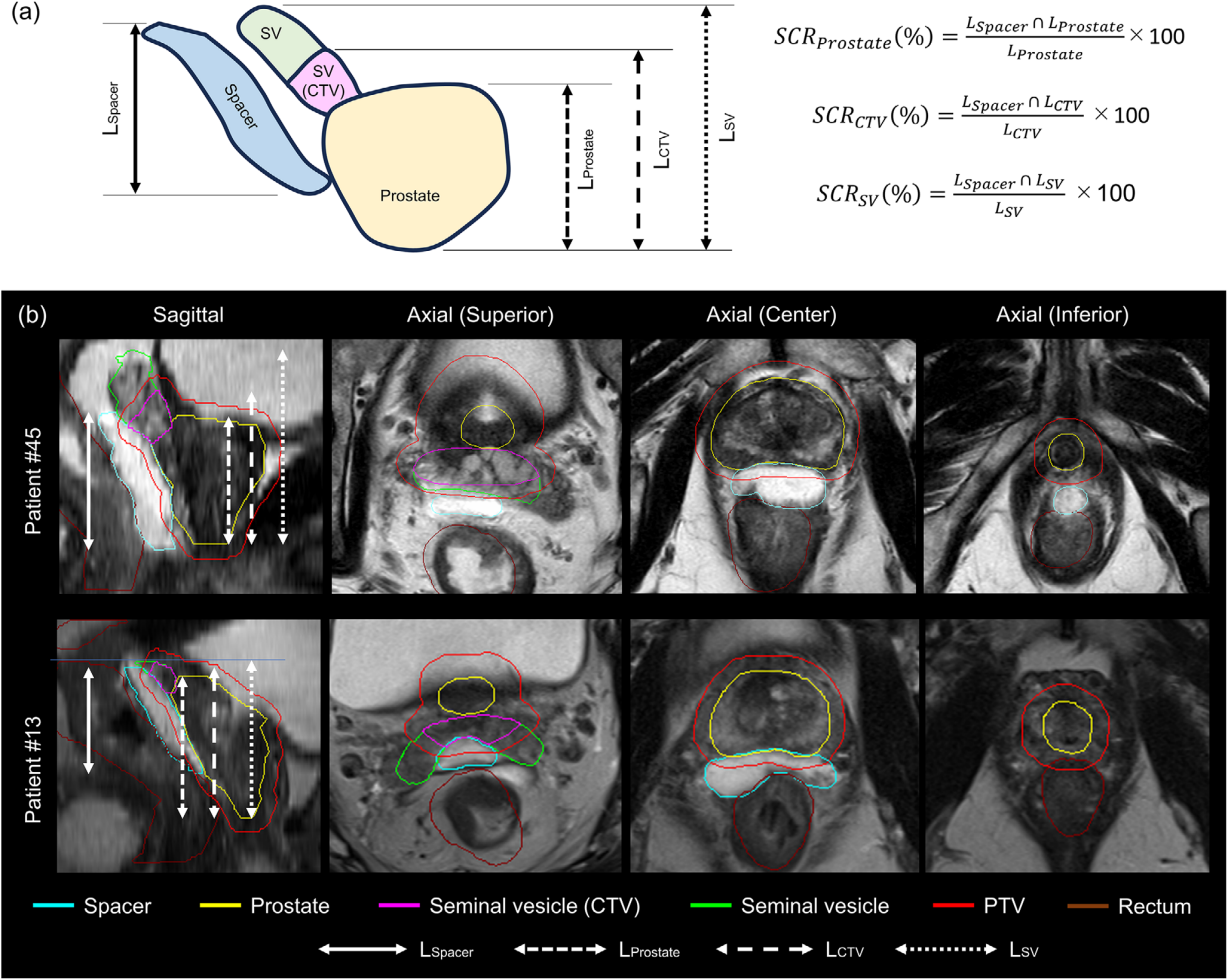
(a) Calculation of SCR for prostate (*SCR*_Prostate_), CTV (*SCR*_CTV_), and prostate plus SV (*SCR*_SV_). (b) Distribution of hydrogel spacer for patients 45 (group 1; *SCR*_Prostate_, *SCR*_CTV_, and *SCR*_SV_ of 100%, 90.6%, and 66.9%, respectively) and 13 (group 2; *SCR*_Prostate_, *SCR*_CTV_, and *SCR*_SV_ of 67.3%, 66.7%, and 62.8%, respectively).

For each patient, the thickness of the hydrogel spacer was manually measured in the axial image at the prostate center, 10 and 20 mm superior to the prostate center (*S*_10mm_ and *S*_20mm_, respectively), and 10 and 20 mm inferior to the prostate center (*I*_10mm_ and *I*_20mm_, respectively). In each axial image, the thickness of the hydrogel spacer was measured at five points: prostate center, 5 and 10 mm to the left of the center (*L*_5mm_ and *L*_10mm_, respectively), and 5 and 10 mm to the right of the center (*R*_5mm_ and *R*_10mm_, respectively). The lengths of the hydrogel spacer (*L*_Spacer_), prostate (*L*_Prostate_), CTV (*L*_CTV_), and prostate plus SV (*L*_SV_) were determined as the distances in the SI direction of the corresponding target volumes (Figure 1a). The spacer cover ratios (SCRs, in percentages) for the prostate (*SCR*_Prostate_), CTV (*SCR*_CTV_), and prostate plus SV (*SCR*_SV_) were calculated by dividing the overlapping length of *L*_Spacer_ and *L*_Prostate_ as well as *L*_CTV_ and *L*_SV_ by *L*_Prostate_, *L*_CTV_, and *L*_SV_ and multiplying by 100, respectively.

The HIQS was calculated as in the method proposed by Liu et al.^12^ The scores for the inserted hydrogel volume (*HIQS*_Vol_), left–right symmetry (*HIQS*_LR_), SI symmetry (*HIQS*_SI_), and mid‐prostate spacing created by the hydrogel (*HIQS*_Spacing_) were calculated as follows:

(1) $$HIQS_{\mathrm{vol}}=\left\{\begin{array}{c} Int\left(V_{\mathrm{Spacer}}\times \left.\frac{25}{16}\right)\;if\;V_{\mathrm{Spacer}}\right.\leq 16\;ml\\ 25\;if\;V_{\mathrm{Spacer}}>16\;ml \end{array}\right.$$

(2) $$HIQS_{\mathrm{LR}}=25-Int\left(\left|\frac{V_{\mathrm{left}}-V_{\mathrm{right}}}{V_{\mathrm{Spacer}}}\right|\times 25\right)$$

(3) $$HIQS_{\mathrm{SI}}=25-Int\left(\left|\frac{V_{\mathrm{Sperior}}-V_{\mathrm{Inferior}}}{V_{\mathrm{Spacer}}}\right|\times 25\right)$$

(4) $$HIQS_{\mathrm{Spacing}}=\left\{\begin{array}{c} Int\left(D_{\mathrm{center}}\times \left.\frac{25}{18}\right)\;if\;\right.D\leq 18\;mm\\ 25\;if\;D>18\;mm \end{array}\right.$$

where *V*_Spacer_ is the volume of inserted hydrogel spacer, *V*_Left_, *V*_Right_, *V*_Superior_, and *V*_Inferior_ are the volumes of the hydrogel space divided by the prostate center, and *D*_center_ is the distance between the prostate and rectum achieved by the hydrogel spacer insertion measured at the center of the prostate. The total HIQS (*HIQS*_total_) was obtained as

(5) $$\begin{array}{ccc} HIQS_{\mathrm{total}} & = & HIQS_{\mathrm{vol}}+HIQS_{\mathrm{LR}}+HIQS_{\mathrm{SI}}\\ & & +\;HIQS_{\mathrm{Spacing}} \end{array}$$

### 2.4 Data analysis

Because the prescribed dose varied depending on the patient, dosimetric parameters for the PTV, rectum, and bladder were evaluated by the relative dose. For the PTV, we obtained dosimetric parameters (in percentages) *D*_max_, *D*_1%_, *D*_50%_, *D*_99%_, and *D*_min_, which indicate the maximum dose, doses to 1%, 50%, and 99% target volume, and minimum dose, respectively. For the rectum and bladder, *D*_max_, *D*_0.03cc_ (dose to 0.03 cc of OAR volume), *D*_1cc_ (dose to 1 cc of OAR volume), *D*_10%_ (dose to 10% OAR volume), *D*_20%_ (dose to 20% OAR volume), and *D*_50%_ (dose to 50% OAR volume) were evaluated.^13^

The patients were divided into two groups. In group 1, the PTV was separated from the rectum, and in group 2, the PTV overlapped with the rectum. The Mann–Whitney *U*, Fisher, or Pearson's chi‐squared test was applied to measure significant differences in patient characteristics, volumes (prostate, SV, CTV, PTV, rectum, and bladder), dosimetric parameters for the target and OARs, thickness of the hydrogel spacer measured at various positions, and values of the hydrogel spacer distribution (i.e., SCR and HIQS) between groups 1 and 2. Value *p* < 0.05 was considered as statistically significant. For parameters with significant differences, the optimal cutoff value for distinguishing between the two groups was determined through receiver operating characteristic analysis. The sensitivity, specificity, and area under the curve (AUC) with 95% confidence intervals (CIs) were calculated. Finally, the relationship between the value with the highest AUC and dosimetric parameters for the rectum was tested using Pearson's correlation coefficient. Pearson's *r* was categorized into very weak (0–0.19), weak (0.2–0.39), moderate (0.40–0.59), strong (0.6–0.79), and very strong (0.8–1). All statistical analyses were performed using SPSS software (version 27; IBM, Armonk, NY).

## 3 RESULTS

Table 1 shows the characteristics of the 29 and 26 patients in groups 1 and 2, respectively. The patient characteristics did not show a significant difference (*p* > 0.05) (Table 1) as well as the volumes of prostate, SV, CTV, PTV, rectum, and bladder between the two groups (Table 2). Table 3 lists the dosimetric parameters for the targets and OARs in the two groups. For the PTV, comparable *D*_max_, *D*_1%_, *D*_50%_, and *D*_min_ were obtained between the groups, while *D*_99%_ in group 1 (97.4 ± 0.6%) was significantly higher (*p* = 0.038) than that in group 2 (96.9 ± 0.8%). For the rectum, *D*_max_ (84.3 ± 10.6%), *D*_0.03cc_ (78.7 ± 11.7%), *D*_1cc_ (60.3 ± 12.1%), and *D*_10%_ (43.7 ± 9.7%) were significantly lower (*p* < 0.05) for group 1 than for group 2 (98.0 ± 4.2%, 94.6 ± 5.1%, 75.3 ± 10.1%, and 51.7 ± 10.1% for *D*_max_, *D*_0.03cc_, *D*_1cc_, and *D*_10%_, respectively). There were no significant differences in the dosimetric parameters of the bladder (*p* > 0.05).

**TABLE 1:** Patient characteristics.

Characteristics	Group 1 (*n* = 29)	Group 2 (*n* = 26)	*p*‐value
Age (*y*), median (range)	69 (60–84)	73 (59–85)	0.133
Risk (*n*)			
Intermediate (favorable)	8	10	0.690
Intermediate (unfavorable)	16	12	
High or Ultra‐high	5	4	
T stage (*n*)			0.202
T1	2	1	
T2	25	25	
T3	2	0	
Gleason Score (*n*)			0.556
3+4	25	22	
4+4	3	4	
5+5	1	0	
PSA (*n*)			0.389
<10	19	20	
10≤	10	6	
Androgen deprivation therapy (*n*)			1.000
Yes	20	17	
No	9	9	
History of Intrapelvic Surgery (*n*)			1.000
Yes	3	2	
No	26	24	
History of pelvic irradiation (*n*)			NA
Yes	0	0	
No	29	26	

**TABLE 2:** Comparison of structure volumes.

	Group 1 (*n* = 29)		Group 2 (*n* = 26)		
Volume (cm^3^)	Mean	SD	Mean	SD	*p*‐value
Prostate	27.6	10.1	29.9	10.9	0.555
Seminal vesicle	12.4	7.4	12.3	6.4	0.840
CTV	32.3	10.4	34.6	12.0	0.686
PTV	70.8	16.3	75.6	19.9	0.590
Rectum	45.0	15.0	46.2	10.8	0.418
Bladder	250.8	80.5	335.0	154.3	0.071

**TABLE 3:** Comparison of dosimetric parameters for PTV and OARs in the two groups; group 1 with the PTV separated from the rectum and group 2 with the PTV overlapping with the rectum.

		Group 1 (*n* = 29)		Group 2 (*n* = 26)		
Dosimetric parameter (%)		Mean	SD	Mean	SD	*p*‐value
PTV	*D*_max_	109.8	1.7	109.2	1.5	0.500
	*D*_1%_	106.3	0.9	106.1	0.9	0.544
	*D*_50%_	102.9	0.4	102.9	0.5	0.893
	^*^ *D*_99%_	97.4	0.6	96.9	0.8	0.038
	*D*_min_	88.7	3.2	86.3	4.6	0.059
Rectum	^*^ *D*_max_	84.3	10.6	98.0	4.2	<0.001
	^*^ *D*_0.03cc_	78.7	11.7	94.6	5.1	<0.001
	^*^ *D*_1cc_	60.3	12.1	75.3	10.1	<0.001
	^*^ *D*_10%_	43.7	9.7	51.7	10.1	0.014
	*D*_20%_	34.4	8.2	38.9	8.1	0.089
	*D*_50%_	22.2	6.5	23.0	5.4	0.438
Bladder	*D*_max_	107.0	1.0	107.1	0.8	0.527
	*D*_0.03cc_	105.8	0.8	105.8	0.7	0.595
	*D*_1cc_	103.9	0.7	104.1	0.6	0.200
	*D*_10%_	72.6	12.9	69.5	15.4	0.601
	*D*_20%_	47.0	15.0	43.8	16.3	0.827
	*D*_50%_	13.6	11.3	11.1	7.3	0.787

* *p* < 0.05.

**FIGURE 2:**
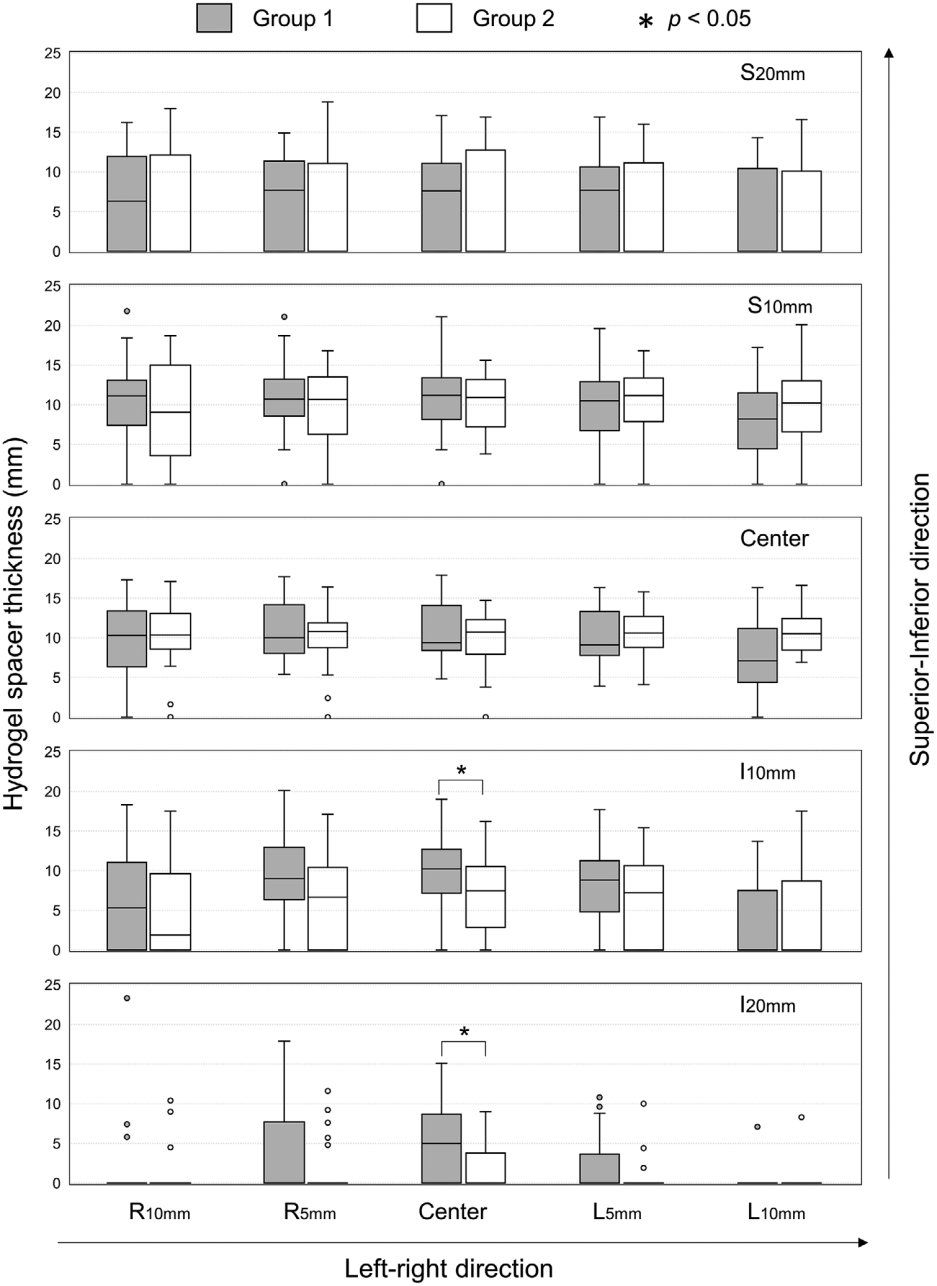
Measured thicknesses of hydrogel spacer at various points.

Figure 2 shows the measured hydrogel spacer thicknesses at various points. The spacer thickness was the highest at the prostate center (1.1 ± 0.4 mm and 1.0 ± 0.4 mm in groups 1 and 2, respectively) and reduced as the spacer moved away in the SI and LR directions. The hydrogel spacer on the inferior side was thinner than that on the superior side. A significant difference in thickness (*p* < 0.05) was observed in the prostate center in *I*_10mm_ (0.9 ± 0.5 mm and 0.6 ± 0.5 mm in groups 1 and 2, respectively) and *I*_20mm_ (0.5 ± 0.5 mm and 0.2 ± 0.3 mm in groups 1 and 2, respectively).

Figure 1b shows the distribution of hydrogel spacer for patients 45 (group 1; *SCR*_Prostate_, *SCR*_CTV_, and *SCR*_SV_ of 100%, 90.6%, and 66.9%, respectively) and 13 (group 2; *SCR*_Prostate_, *SCR*_CTV_ and *SCR*_SV_ of 67.3%, 66.7%, and 62.8%, respectively). Table 4 lists the values of hydrogel spacer distribution between the two groups. In both groups, equivalent volumes of hydrogel spacer were inserted (11.4 ± 3.0 cc and 11.2 ± 2.8 cc in groups 1 and 2, respectively; *p* = 0.505). *SCR*_Prostate_ (89.5 ± 12.2%), *SCR*_CTV_ (86.1 ± 11.7%), and *SCR*_SV_ (76.1 ± 11.9%) in group 1 were significantly higher (*p* < 0.05) than those in group 2 (74.7 ± 10.3%, 72.5 ± 9.8%, and 65.4 ± 11.8% for *SCR*_Prostate_, *SCR*_CTV_, *SCR*_SV_, respectively). In contrast, the HIQS values (*HIQS*_Vol_, *HIQS*_LR_, *HIQS*_SI_, *HIQS*_Spacing_, and *HIQS*_total_) did not provide significant differences between the two groups (*p* > 0.05). Table 5 lists the cutoff values, sensitivities, specificities, and AUCs for distinguishing between the two groups. The highest sensitivity of 89.7% was obtained at 64.4% cutoff for *SCR*_SV_. Overall, the AUC (0.822; 95% CI, 0.708–0.936) was the highest for *SCR*_Prostate_ at 93.6% cutoff, 62.1% sensitivity, and 100% specificity.

**TABLE 4:** Comparison of quantitative values of hydrogel spacer distribution between two groups.

	Group 1 (*n* = 29)		Group 2 (*n* = 26)		
Quantitative value	Mean	SD	Mean	SD	*p*‐value
Spacer volume (cc)	11.4	3.0	11.2	2.8	0.505
^*^SCR_Prostate_ (%)	89.5	12.2	74.7	10.3	<0.001
^*^SCR_CTV_ (%)	86.1	11.7	72.5	9.8	<0.001
^*^SCR_SV_ (%)	76.1	11.9	65.4	11.8	0.003
HIQS_Vol_	17.2	4.8	17.0	4.5	0.565
HIQS_LR_	19.9	4.2	19.7	5.1	0.819
HIQS_SI_	15.6	5.7	13.9	6.8	0.299
HIQS_Spacing_	14.5	5.1	13.4	4.8	0.774
HIQS_total_	67.3	12.1	64.1	12.2	0.453

* *p* < 0.05.

**TABLE 5:** Cutoff values, sensitivities, specificities, and AUCs for distinguishing between the two groups derived from receiver operating characteristic analysis.

Parameter	Cutoff value (%)	Sensitivity (%)	Specificity (%)	AUC (95% CI)
SCR_Prostate_	93.6	62.1	100.0	0.822 (0.708–0.936)
SCR_CTV_	77.8	75.9	76.9	0.807 (0.690–0.924)
SCR_SV_	64.4	89.7	57.7	0.737 (0.602–0.873)

**FIGURE 3:**
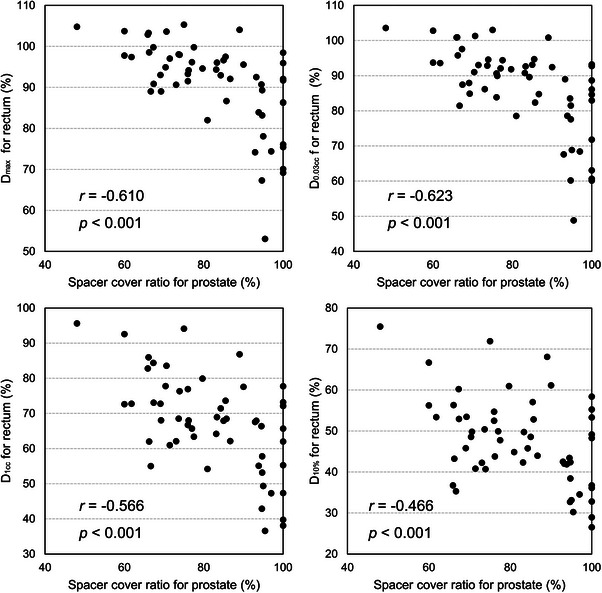
Relationship between SCR and dosimetric parameters for prostate.

Figure 3 shows the relationship between *SCR*_Prostate_ and the dosimetric parameters for the rectum. A significantly strong correlation was observed between *SCR*_Prostate_ and *D*_max_ (*r* = 0.610, *p* < 0.001) and *D*_0.03cc_ (*r* = 0.623, *p* < 0.001). In other words, a higher *SCR*_Prostate_ indicates a reduced high‐dose radiation to the rectum.

## 4 DISCUSSION

We explored quantitative parameters of hydrogel spacer distributions to separate the rectal volume from the PTV in linear‐accelerator‐based SBRT for prostate cancer. In conventional fractionated radiotherapy for prostate cancer (prescribed dose of 70−79.2 Gy), using a hydrogel spacer is rare, thus resulting in overlapping PTV and rectum. Fiorino et al. reported that dosimetric parameter *V*_70Gy_ (i.e., relative volume receiving 70 Gy) for the rectum should remain in 25%−30% to maintain the incidence of moderate/severe rectal bleeding below 5%−10%.^14^ Therefore, treatment plans should reduce the high‐dose region of the rectum within the PTV, being inconsistent with guaranteeing an adequate dose to the PTV. In SBRT, the rectum is strictly constrained to minimize rectal toxicity. The dose constraint for the rectum was set as *D*_max_ of 38 Gy with a prescribed dose of 38 Gy in four fractions at the University of North Carolina (NCT 00643617). Wang et al. stated that toxicity may be reduced by limiting *D*_max_ for the rectum to the prescribed dose.^13^ To maintain *D*_max_ for the rectum below the prescribed dose without compromising the dose delivered to the PTV, the rectum should be physically separated from the PTV using a hydrogel spacer.

A hydrogel spacer is inserted into the rectoprostatic space, and its material remains stable in size for several months until its absorption. Wang et al. proposed a method to predict rectal dose from the anatomical shape of the rectum and PTV.^15^ Paetkau et al. found that the volumes of rectum in PTV as well as CTV and rectum volumes offered highest correlation with rectal doses.^16^ Characterizing the quality of the hydrogel spacer distribution is an active research area. Grossman et al. proposed a spacer quality score for the prostate‐rectal interspace in a range from 0 to 2 based on the thickness of the hydrogel spacer at various points, and they found that the score was significantly associated with dosimetric parameters *D*_max_ and *D*_1cc_ for the rectum.^17^ The HIQS was developed by Liu et al.^12^ as an innovative indicator that quantifies the distribution of spacers and it might provide insights into physicians learning and dosimetric outcome. However, a correlation between the HIQS and overlapping between the PTV and rectum was not determined in linear accelerator based SBRT for prostate cancer. As the HIQS was originally intended for low‐dose‐rate brachytherapy, it may not have been significantly correlated with the studied dose distribution of volumetric modulated arc therapy. Such quantitative measurements of hydrogel spacer distribution are labor intensive in clinical practice and may hinder goal achievement to physicians who insert spacers. Hwang et al. demonstrated that patients with the lower D_max_ of rectum and larger values of angle θ formed by the prostate, hydrogel, and rectum multiplied by hydrogel spacer volume did not show the rectal toxicity.^11^ Because our proposed simplified *SCR*_Prostate_ is associated with higher doses to the rectum, it may be a useful indicator in predicting rectal toxicity in clinical practices.

The insertion of spacers with uniform thickness is not easy in clinical practice. The spacer thickness decreased with increasing distance from the prostate center, and this trend was greater in the inferior than in the superior direction (Figure 2). Eckert et al. reported similar results, with the distance between the prostate and rectum after hydrogel spacer insertion being larger in the middle and base (superior direction) of the prostate than in the apex (inferior direction).^18^ Whalley et al. observed the area of radiation proctitis at the prostatic apex level in one patient, and the hydrogel spacer provided adequate separation except for the apex region.^19^Our suggested parameter *SCR*_Prostate_, which represents the hydrogel spacer distribution, was significantly correlated with the dosimetric parameters for the rectum (Figure 3) and may be a simple indicator for physicians. In this study, if more than 93.6% of the prostate posterior could be covered by hydrogel spacer, there was no overlap between PTV and rectum in all cases (Table 5). Therefore, when inserting hydrogel spacer, care should be taken to ensure that the apex region is also adequately covered. In general, a needle tip is placed at the center of the prostate, and the hydrogel spacer is then injected. Fukumitsu et al. presented a hydrogel injection technique, in which the needle was placed at a level corresponding to a ratio base: apex side of 6:4, and separation was observed at all levels from the base to the apex of the prostate.^20^ Insufficient separation of the rectum and prostate may result in high‐dose irradiation to the rectum. Thus, the entire prostate, from its base to apex, must be covered with a hydrogel spacer.

This study has various limitations. First, the number of patients was small, possibly affecting the statistical calculations. Second, a wider distribution of spacers on the SI direction was more effective in reducing rectal dose than a thicker insertion of spacers.at a given point when using a CTV‐to‐PTV margin of 3 mm in the posterior direction was adopted, but the results may vary depending on the posterior margin size. Studenski et al. demonstrated that a posterior margin of 3 mm in prostate SBRT allowed to maintain prostate coverage while compromising the PTV coverage.^21^ Third, inter‐observer variability occurred in prostate and spacer contouring, possibly affecting our results. Fourth, the relationship between *SCR*_Prostate_ and rectal toxicity could not be investigated, and further studies are needed to determine whether *SCR*_Prostate_ can be used to predict rectal toxicity. Because of inter‐ and intra‐fractional motion of prostate and rectum during actual treatment, the rectal dose may differ between treatment planning and treatment. It might be necessary to develop an alternative index to *SCR*_Prostate_ that is less sensitive to inter‐ and intra‐fractional motion. Finally, the Barrigel hydrogel spacer (Palette Life Sciences, Stockholm, Sweden), which can take time to sculpt from the base to apex because of the lacking polymerization,^22^, ^23^ and SpaceOAR Vue (Boston Scientific), which consists of a hydrogel covalently bonded with iodine to improve visibility in CT images,^24^ are also available. The difficulty in inserting a hydrogel spacer in the proper position may vary depending on the type of hydrogel spacer, but this was not assessed in our study.

In conclusion, the separation between the PTV and rectum is a significant indicator for reducing rectal dose in SBRT for prostate cancer. The hydrogel spacer is significantly thinner in the inferior direction (apex) in group 2 (overlapping PTV and rectum) than in group 1 (without overlapping). *SCR*_Prostate_ seems to be an adequate indicator for distinguishing between the two groups and may assist physicians in the appropriate insertion of a hydrogel spacer.

## AUTHOR CONTRIBUTIONS

All authors participated in the writing of this article and are responsible for its content. The authors declare that the contents of this manuscript have neither been published nor submitted for publication elsewhere.

## CONFLICT OF INTEREST STATEMENT

Shingo Ohira, Masanari Minamitani, and Keiichi Nakagawa belong, is an endowment department, supported with an unrestricted grant from Elekta K. K. However, the sponsor had no role in this study.
